# Nation-building policies in Timor-Leste: disaster risk reduction, including climate change adaptation

**DOI:** 10.1111/disa.12082

**Published:** 2014-09-05

**Authors:** Jessica Mercer, Ilan Kelman, Francisco do Rosario, Abilio de Deus de Jesus Lima, Augusto da Silva, Anna-Maija Beloff, Alex McClean

**Keywords:** climate change adaptation, disaster risk reduction, nation-building, small island developing states, Timor-Leste

## Abstract

Few studies have explored the relationships between nation-building, disaster risk reduction and climate change adaptation. Focusing on small island developing states, this paper examines nation-building in Timor-Leste, a small island developing state that recently achieved independence. Nation-building in Timor-Leste is explored in the context of disaster risk reduction, which necessarily includes climate change adaptation. The study presents a synopsis of Timor-Leste's history and its nation-building efforts as well as an overview of the state of knowledge of disaster risk reduction including climate change adaptation. It also offers an analysis of significant gaps and challenges in terms of vertical and horizontal governance, large donor presence, data availability and the integration of disaster risk reduction and climate change adaptation for nation-building in Timor-Leste. Relevant and applicable lessons are provided from other small island developing states to assist Timor-Leste in identifying its own trajectory out of underdevelopment while it builds on existing strengths.

## Introduction

In the context of new sovereignty, one definition of nation-building includes the involvement of internal and external actors 'as part of a broader effort to promote political and economic reforms with the objective of transforming a society emerging from conflict into one at peace with itself and its neighbors' (Dobbins et al., 2007, p. 21). Essential components of any nation-building include military and police contingents, civil administrators and experts on political reform and economic development.[2] These components aim to undertake nation-building tasks such as human security, humanitarian relief, governance, economic stabilisation, democratisation and development (Dobbins et al., 2007).

Nation-building faces significant challenges, both in the theory of trying to define a nation and national identity, and in the practice of maintaining momentum in improving citizens' lives without being derailed from the dream of successful independence due to enduring development concerns.[3] Further complications arise given the fact that nation-building requires an integration of national and international efforts. That is needed to coordinate the support and the interventions of major powers, financial donors and neighbouring countries to avoid duplication and

*Disasters,* 2014, 38(4): 690–718. © 2014 The Author(s). *Disasters* © Overseas Development Institute, 2014
This is an open access article under the terms of the Creative Commons Attribution License, which permits use, distribution and reproduction in any medium, provided the original work is properly cited.
Published by John Wiley & Sons Ltd, 9600 Garsington Road, Oxford, OX4 2DQ, UK and 350 Main Street, Malden, MA 02148, USA

competition over nation-building efforts and resources (Chernilo, 2007; Dobbins et al., 2007).

Given these challenges, some smaller jurisdictions opt to avoid independence because they wish to preserve their formal connections to their governing state and because, as many argue, the jurisdiction would not survive without aid and support from the governing state (Baldacchino and Milne, 2006; 2009). Disasters are among the development concerns that can threaten citizens' well-being—be it over the short term, due to earthquakes or hurricanes, or over the long term, due to persistent droughts and climate change impacts.[4]

Interactions between various forms of disaster and sovereignty have long been studied. Nel and Righarts (2008) reach back to Sparta in 465–64 BC, identifying an earthquake disaster as seeding a revolt by slaves for their freedom. Lewis (1999, p. 25) argues that '[t]he November 1970 cyclone, and its subsequent alleged mismanagement, was one of the many influences that triggered the Bangladesh War of Independence which commenced in March 1971'. Glantz (1976) details the politics of addressing the Sahel drought in the wake of newly independent states across sub-Saharan Africa. Various arguments indicate how dealing with disaster both solidified support for some post-independence governments and undermined efforts by others in nation-building, depending on the disaster impacts in combination with non-disaster factors.

Meanwhile, more recent disaster diplomacy studies examine how disasters and disaster risk reduction (DRR) rarely support peace processes, including conflicts about independence (Kelman, 2012). For example, the 26 December 2004 tsunami devastated parts of Sri Lanka that were the core areas of support for a 30-year separatist armed struggle. The tsunami's aftermath perpetuated and bolstered the conflict, and the Sri Lankan government eventually succeeded with a military solution to the conflict (Kelman, 2012).

Disaster diplomacy theory, which suffers from gaps in the literature, does not explicitly address nation-building factors that influence disaster response and disaster risk reduction—or vice versa. Overall, few studies have examined how disaster-related activities can become an integral part of nation-building or how a specific disaster might undermine or boost nation-building efforts. Similarly, in practice, the main international blueprint for disaster risk reduction—the Hyogo Framework for Action (UNISDR, 2005)—mentions neither state-building nor nation-building. Further research could enhance the general understanding of the interactions between nation-building and disaster risk reduction, both in theory and in practice.

One geographical area about which some case studies exist is the small island developing states (SIDS). SIDS are a collection of 52 islands and territories in the tropics and sub-tropics. Their vulnerability and resilience to disasters, and especially climate change, has been well articulated in theory[5] and in practice.[6] This literature emphasises that SIDS characteristics display both vulnerabilities and resiliencies to different forms of disaster and each needs to be factored into any plans for improvements.

Although the literature on nation-building and disasters is limited, a few SIDS have been researched in this context. In Niue in 2004, Cyclone Heta prompted some to close their small businesses and to emigrate to New Zealand with their families, reducing prospects for full independence (Hermann et al., 2005). Meanwhile, research suggests that climate change impinges on the sovereignty and viability of low-lying atolls, with some authors emphasising a bleak future (Barnett and Adger, 2003); meanwhile, others seek solutions to the identified challenges, even if climate change adaptation (CCA) involves migration with the aim of setting up an independent state elsewhere (Kelman, 2006).

One SIDS that recently achieved independence is Timor-Leste (East Timor), formerly an occupied area of Indonesia. The objective of this paper is to examine nation-building in Timor-Leste in the context of DRR, which necessarily links to CCA (Shaw, Pulhin and Pereira, 2010a; 2010b). Following the spirit of nation-building and the necessary involvement of national and international actors, this study and its recommendations emerge from a combination of national and international perspectives. The authors include three national government colleagues who were directly involved in DRR, including CCA, at a policy level; three international colleagues who have worked within civil society and United Nations agencies in Timor-Leste on the topics of DRR, including CCA, for extended periods of time; and one colleague whose work has mainly taken a SIDS and DRR (and CCA) perspective. The discussion presents Timor-Leste as a case study of a newly independent state that implements DRR, including CCA, in the context of nation-building tasks, including governance and development, with a specific focus on the national level.

While CCA covers activities to reduce the impacts of projected climate change, discussing DRR, including CCA, necessarily involves climate change mitigation, such as activities to reduce climate change-related gas emissions and to increase sinks for these gases (IPCC, 2007). IPCC (2007) and others separate CCA and climate change mitigation, although scientists have long called for them to be connected and integrated (Dang, Michaelowa and Tuan, 2003; Kane and Shogren, 2000; Mills, 2007). Similarly, many DRR activities are strongly linked to energy efficiency (Begum, Komoo and Pereira, 2011; Etkin, 2008) and sustainable forestry (Sudmeier-Rieux and Ash, 2009; Wisner, 2001), both of which are key climate change mitigation activities. While highlighting CCA, this paper discusses both CCA and climate change mitigation within the context of DRR.

The next section provides background on the independence struggle and disaster history of Timor-Leste. Then, the state of knowledge in Timor-Leste regarding DRR, including CCA, is reviewed within the context of nation-building. Current laws, policies and development-related endeavours relevant to DRR, including CCA, and how they have evolved and stalled, are summarised. Finally, the recommendations and conclusion sections compare Timor-Leste to other SIDS in the context of academic literature on similar topics, including other SIDS case studies, while discussing possible trajectories that Timor-Leste could select. This paper is presented in the context of theories and practices regarding the interactions between disasters and politics.

## Timor-Leste as a small island developing state, its history, and its nation-building efforts

Timor-Leste is the eastern half of the island of Timor, situated in the Indonesian archipelago in South East Asia (see Figure 1). The country includes the exclave Oecusse to the west and two small offshore islands, Atauro and Jaco. The western half of the island of Timor is West Timor, which belongs to Indonesia. Timor-Leste covers a total area of 14,874 km² and is divided into 13 districts, with a population of just over 1 million, as of the last census undertaken in 2010. About 43% of the population resides in just three districts: Dili, Baucau and Ermera; the capital, Dili, has a population of about 200,000 (GoTL, 2010).

A small country with a difficult history, Timor-Leste suffered more than 400 years of colonialism and foreign occupation by the Portuguese. On 28 November 1975, Timor-Leste declared independence from Portugal, only to be invaded and occupied by Indonesia nine days later. In July 1976, Timor-Leste was incorporated into Indonesia as the province of 'Timor Timur' and was occupied for a further 24 years, during which the struggle for independence continued. In the ongoing conflict, at least 102,800 conflict-related deaths occurred between 1974 and 1999 (Romesh and Ball, 2006).

On 30 August 1999, Timor-Leste's citizens voted in an Indonesian-initiated referendum that presented the choice between independence and limited autonomy within

**Figure 1** Timor-Leste

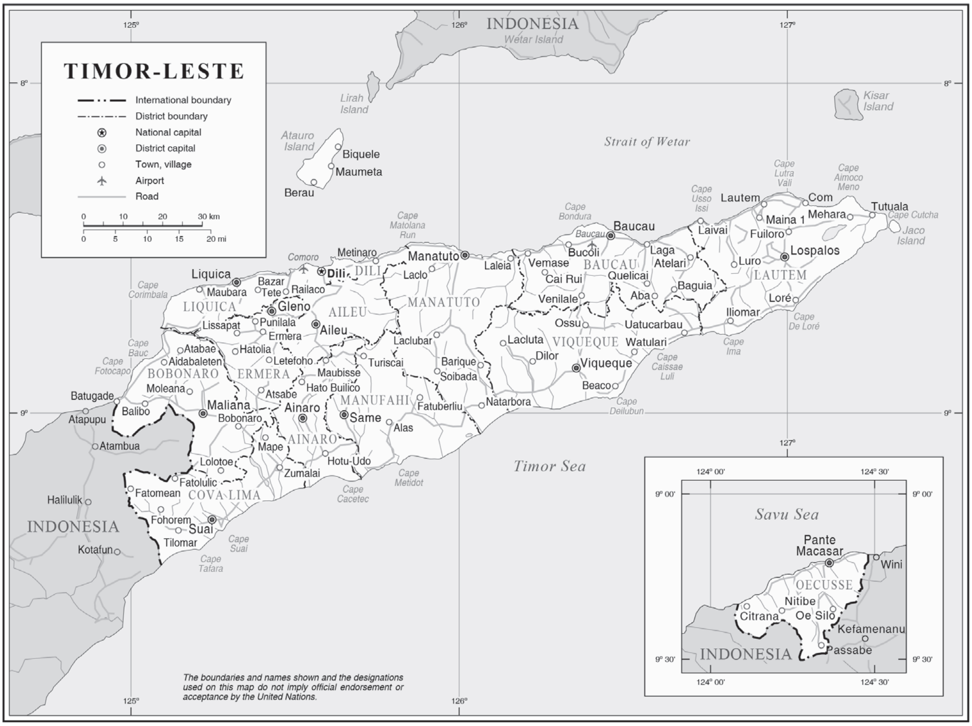

Map No. 4111 Rev. 11 UNITED NATIONS
November 2011 (Colour)

Department of Field Support
Cartographic Section

**Source:** UN (2011).

Indonesia. With a strong majority of approximately 78%, independence was favoured (Hill, 2001). This outcome triggered a strong reaction from those in Timor-Leste and Indonesia who opposed independence. The result was a three-week campaign of violence and destruction known as 'Operation Clean Sweep', in which Indonesian armed forces and locally organized militias reduced buildings to rubble and executed hundreds, if not thousands, of East Timorese. More than three-quarters of the country's then population of 890,000 were displaced and 70% of the infrastructure in Timor-Leste was destroyed as streets were burned one by one (Chopra, 2002).

Timor-Leste achieved independence at considerable expense, but most significant was the loss of personnel and knowledge needed to rebuild (Chopra, 2002). The three-week campaign of violence ended in September 1999 with the deployment of the Australian-led International Task Force in East Timor. Plans for an orderly transfer of power were abandoned and the United Nations Transitional Administration in East Timor replaced all pre-existing authorities in the territory, becoming the formal governing body. Following this transition period, on 20 May 2002, Timor-Leste was internationally recognised as an independent state.

In the decade since gaining independence, Timor-Leste has faced significant challenges in nation-building. Initially lauded for successfully building a new independent identity, Timor-Leste was again marred by violence in 2006, as deep-seated social tensions rose to the surface,[7] and in February 2008, when Timor-Leste's president and prime minister were nearly assassinated in a coup attempt. In addition to conflict and social tensions, including gender-based violence, as a small island developing state, Timor-Leste faces considerable development challenges in terms of its mountainous terrain and geology, relative isolation, natural hazards, dependence on agriculture and high levels of unemployment (Hill, 2001; Méheux, Dominey-Howes and Lloyd, 2007; Pelling and Uitto, 2001; Sandlund et al., 2001). These challenges are compounded by poor natural resource management, including unsustainable farming and fishing practices and an overdependence on oil and gas exploitation.

Despite the country's natural resource wealth of oil and gas (Steele, 2002), more than 70% of its inhabitants reside in rural areas and depend on agriculture for their livelihood (GoTL, 2010). Timor-Leste is prone to a wide range of natural hazards, including droughts, floods, landslides, earthquakes and tsunamis, in addition to the threat of climate change (Norton and Waterman, 2008). The latter's effects include seasonal weather fluctuations that influence agriculture as well as new impacts such as sea-level rise and ocean acidification (Barnett, Dessai and Jones, 2007; Kirono, 2010; Wasson, 2001).

However, given the many years of conflict, there is a lack of credible data sets in Timor-Leste as information has either not been recorded or has been lost or destroyed (Norton and Waterman, 2008). This is demonstrated by the differences in hazard history data recorded by EM-DAT (see Table 1) and DesInventar (see Table 2). Results from both databases outline some limited history on disasters recorded in Timor-Leste, but this is far from a complete picture, with many localised events going unrecorded. Despite these challenges, Timor-Leste as a decade-old state has a huge opportunity to

**Table 1** Recorded hazards in Timor-Leste since independence, as reported by EM-DAT, 2002–13

| | 2003 | 2005 | 2006 | 2007 | Total |
|---|---|---|---|---|---|
| Drought | | | | 1 | **1** |
| Epidemic | | 1 | | | **1** |
| Flood | 2 | | | 2 | **4** |
| Storm | | | 1 | | **1** |
| **Total** | **2** | **1** | **1** | **3** | **7** |

**Note:** No data was recorded after 2007.

**Source:** EM-DAT (n.d.)

**Table 2** Recorded hazards in Timor-Leste since independence, as reported by DesInventar, 2002–13

| Event | 2002 | 2003 | 2004 | 2005 | 2006 | 2007 | 2008 | 2009 | 2010 | 2011 | 2012 | 2013 | Total |
|---|---|---|---|---|---|---|---|---|---|---|---|---|---|
| Accident | | 2 | | | | | | | 4 | 1 | | 1 | **8** |
| Conflict | 2 | | 1 | | 2 | 5 | | 1 | | 3 | 1 | 1 | **16** |
| Drought | | 1 | | | | | | | | | | | **1** |
| Epidemic | | | | | | | | | | 1 | | | **1** |
| Fire | 2 | 8 | 4 | | | | 1 | 40 | 20 | 29 | 55 | 22 | **181** |
| Flood | 1 | 10 | | | 1 | 7 | 4 | 7 | 88 | 36 | 25 | 75 | **254** |
| Landslide | | 1 | | | | | | 4 | 16 | 12 | 9 | 6 | **48** |
| Strong wind | 3 | 3 | 5 | | 3 | 3 | 12 | 32 | 93 | 47 | 74 | 33 | **308** |
| **Total** | **8** | **25** | **10** | **0** | **6** | **15** | **17** | **84** | **221** | **129** | **164** | **138** | **817** |

**Source:** DesInventar (n.d.)

learn from past experience around the world regarding nation-building efforts—including the internal capabilities needed for dealing with disasters and climate change, as discussed below.

## Disaster risk reduction, climate change adaptation and the state of knowledge in Timor-Leste

As a relatively new state, Timor-Leste is still in the process of building its policies and institutional structures for DRR, including CCA (Barbosa, 2006). Within Timor-Leste's government, the understanding of disaster risk, climate change and potential impacts on the country's development is growing. Yet severe gaps in resources, capacities and data are impeding the incorporation of risks into national and, subsequently, local-level development planning (NDES, 2007a; 2007b; Norton and Waterman, 2008).

## Disaster risk reduction in Timor-Leste

In Timor-Leste, responsibilities for addressing disaster risk are situated within the Ministry of Social Solidarity and are centred around the National Disaster Management Directorate (NDMD) in Dili. The NDMD's mission is 'to consolidate a culture of prevention and to provide the Nation with means to prevent natural disasters and/or at least to minimize the effects of disasters' (NDMD, 2008, p. 2). In the Fourth Constitutional Government Program of the Council of Ministers for 2007–12, the government states that the core functions of the NDMD are to: 'a) promote studies of the identification of risk zones; b) create early warning systems, particularly related to rains and drought; c) conduct training and capacity building of human resources in the area of disaster risk management; d) be able to provide immediate response when disaster occurs; and e) establish inter-sectoral coordination mechanisms to respond to natural disasters' (NDMD, 2008, p. 2).

In 2008, with support from various stakeholders, Timor-Leste's National Disaster Risk Management Policy (NDRMP) was finalised. The NDRMP provided a platform from which to develop programmes and plans for DRR in Timor-Leste in 2008–12 (NDMD, 2008). In line with internationally recognised approaches to DRR, notably the Hyogo Framework for Action, to which Timor-Leste is a signatory, the NDRMP aims to move beyond traditional disaster response modes towards proactive DRR. As outlined in the NDRMP, the Inter-Ministerial Commission for Disaster Management (CIGD) was set up to guide NDRMP implementation and ensure coordination across line ministries. However, after a meeting held in 2009, the CIGD did not meet for at least two years due to limited participation from other sectoral ministries.

The NDRMP also outlines the structure for disaster management committees at the district level, with each of the 13 districts in Timor-Leste having a district disaster management committee (DDMC) operating under the relevant district government (see Figure 2). Decisions are decentralised to DDMCs during disasters but, in reality, given the lack of resources and capacity, decisions are still directed back through the NDMD and subsequently to the Secretary of State for Social Assistance and National Disasters and the Ministry of Social Solidarity, whose involvement is compulsory if the number of fatalities exceeds 21 persons. Complicating matters is the fact that district administrators are designated as district disaster coordinators, DRR responsibilities become second in priority, after the normal administrative duties; this trend is only reinforced given that DDMCs fall under the Ministry of Interior (although this structure is currently under review). The 13 DDMCs are further sub-divided into sub-district disaster management committees as well as *suco* (town/village) and *aldeia* (sub-village) disaster management committees (see Figure 2).

While such decentralisation should assist in the provision of an overall, coherent and transparent structure for a national DRR strategy with clear chains of command, it is hindered by a lack of capacity and resources at all levels. In addition, there is an absence of specific legal frameworks and laws outlining the mandates of each of these bodies in relation to DRR (NDMD, 2010). For example, at the district, sub-district

**Figure 2** National disaster risk management system of Timor-Leste

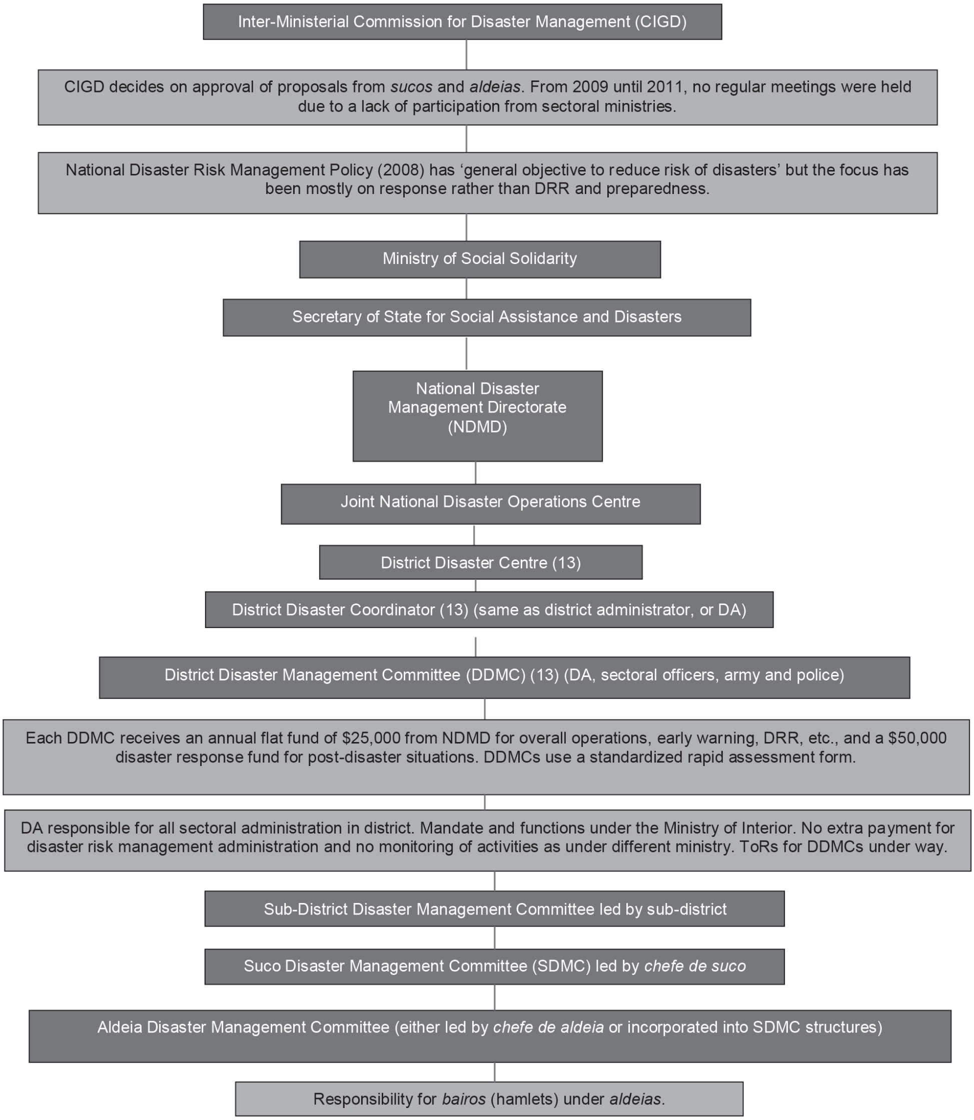

**Note:** Light shading is applied to descriptions whereas darker shading is applied to actual committee names.
**Source:** adapted from Beloff (2011).

and *suco* levels, there is a clear lack of understanding of the NDRMP, the success of which depends on the collaboration and coordination of a large number of stakeholders, given the absence of any laws.

Currently, less than 1% of the national budget is allocated to DRR and there has been limited success in setting up the CIGD to oversee implementation of the NDRMP. While non-specific expenses may be contributing to DRR although they are not identified as DRR, a limited understanding of the interconnectedness of DRR with other development sectors has produced a noticeable lack of commitment from line ministries. For example, the recently developed Strategic Development Plan 2011–2030 for Timor-Leste has not explicitly reflected nor integrated DRR as one of its development priorities. As a result of the limited success of the CIGD and to facilitate discussions with other stakeholders, especially civil society, the NDMD established a Community-based Disaster Risk Management Working Group with monthly meetings. The group helps to coordinate the work of all stakeholders in strengthening community resilience[8] and supporting and developing government structures—especially district, sub-district and *suco*-level disaster management committees—to implement DRR strategies and policies (NDMD, 2010).

## Climate change adaptation and mitigation in Timor-Leste

The National Directorate of Meteorology and Geophysics (NDMG) is responsible for collecting and analysing meteorological and geophysical data. The expansion of climate data monitoring is seen as a high priority (Barbosa, 2006). Currently, only limited data is available on observed climate variability and trends for Timor-Leste (Barnett, Dessai and Jones, 2007; Kirono, 2010; Wasson, 2001). To address this gap, the NDMG is currently working with the Pacific Climate Change Science Program to re-establish a meteorological network, to develop professional capacity and to recover, digitise and analyse data collected under the Portuguese and Indonesian administration periods (Da Silva and Moniz, 2010).

Using results of studies undertaken in Australia, Indonesia and across Asia, Kirono (2010) indicates that under climate change, Timor-Leste will experience a) increased temperatures; b) increased rainfall, with extreme rainfall events projected to become fewer but more intense; c) increased sea surface temperatures; d) sea level rise; e) increased ocean acidification; f) a likely increase in the inter-annual variability of the Asian monsoon; and g) increased and/or exacerbated hazard events, including flooding, landslides, storms and drought. Given that Timor-Leste depends heavily on agriculture, the country is extremely vulnerable to these impacts. However, there is limited awareness of climate change impacts and of the policies and actions needed to reduce greenhouse gas emissions and to adapt (NDES, 2007a; 2007b).

While CCA is a cross-sectoral issue, as is DRR, responsibilities for climate change currently reside within the Secretariat for Environment in the Ministry of Economy and Development in Timor-Leste. At the national level, two main initiatives stand out with respect to climate change. First, on 8 January 2007, Timor-Leste signed the United Nations Framework Convention on Climate Change (UNFCCC), under which it is required to develop and submit a national communications document (currently under way) (UNDP, 2009). In addition, as a least developed country within the UNFCCC, Timor-Leste has produced a National Adaptation Programme of Action (NAPA), in which the Ministry for Social Solidarity and the NDMD are key stakeholders. Table 3 presents a summary of priority adaptation activities identified in the NAPA.

The NAPA for Timor-Leste was produced through consultation with five districts that were considered representative of Timor-Leste's environmental and climatic

**Table 3** Summary of priority adaptation options identified in Timor-Leste's NAPA

| Rank | Adaptation options | Activities |
|---|---|---|
| 1. | Food security | • Integrated agro-forestry and watershed management.<br>• Integrated sustainable land management.<br>• Reforestation of degraded land.<br>• Improvement of physical infrastructure, civil engineering and natural vegetation methods to prevent landslides.<br>• Education and awareness raising on sustainable agriculture and forest management. |
| 2. | Water resources | • Building of infrastructure to protect water sources.<br>• Enhancement of government and community strategies on drought response.<br>• Creation and enhancement of the water harvesting model and distribution system.<br>• Control of the quantity of water used by industry and water pollution. |
| 3. | Human health | • Strengthening of integrated community health services.<br>• Strengthening of the integrated early warning system at the community level.<br>• Review of existing guidance and standards issued by the Ministry of Health on respiratory, airborne and vector diseases. |
| 4. | Natural disasters | • Establishment of early warning systems.<br>• Integration of climate risk information into traditional DRR and disaster risk management. |
| 5. | Forests, biodiversity and coastal ecosystems resilience | • Maintenance of mangrove plantations and awareness raising to protect coastal ecosystems.<br>• Inclusion of ecosystem management in national planning. |
| 6. | Livestock production | • Improvement of planning and the legal framework to promote sustainable and balanced food for livestock production. |
| 7. | Physical infrastructure | • Review of existing laws, regulations and standards to enhance resilience of critical infrastructure.<br>• Passing of new legislation to strengthen and guarantee national development through improved regulations. |
| 8. | Oil and gas sector | • Protection of offshore infrastructure against strong wave damage that could affect the distribution of gas and oil, and a reduction of accidents and destruction of offshore oil and gas infrastructure. |
| 9. | National institutional capacity development for climate change | • Strengthening of the mandate of the cross-sectoral national climate change team to improve coordination and engagement.<br>• Establishment of the Climate Change Unit.<br>• Capacity development support for key non-governmental institutions.<br>• Development of a national climate change strategy and action plan.<br>• Promotion of sub-national capacity development for improved adaptation planning and implementation.<br>• Strengthening of the hydro-meteorological department in the NDMG to collect, compile, analyse and disseminate climate-related data. |

**Source:** adapted from MED (2010)

conditions. While time and financial constraints limited the engagement of further districts for the NAPA process, those involved in the process acknowledge that future studies would benefit from a countrywide consultation. This would provide further information on the historical[9] and current experiences of climate variability and change within Timor-Leste. Such contributions are especially important given the lack of historical meteorological data for Timor-Leste. There is also a clear lack of understanding within all 13 districts of the scientific reasons for climate change and potential long-term impacts (Oxfam, 2011).

Regarding climate change mitigation, on 14 October 2008, Timor-Leste ratified its accession to the UNFCCC's Kyoto Protocol, which came into force in the country on 12 January 2009. Every other SIDS with the authority—the 38 independent states along with the Cook Islands and Niue, whose status is full self-government in free association with New Zealand—ratified and put into force the Kyoto Protocol by July 2008. Timor-Leste was the last SIDS to complete the process. None of these SIDS, however, is listed as an 'Annex I' country, on which the burden of emissions reductions falls under the Kyoto Protocol.

Nonetheless, three SIDS—the Maldives, Niue, and Tuvalu—had committed to becoming carbon neutral by 2020 under the United Nations Environment Programme's Climate Neutral network, which closed in 2011 (UNEP News Centre, 2011). The mandates are not always clear as to how externally linked emissions, such as those related to the shipping and aviation industries, are factored into the calculations. Similarly, several SIDS—such as Trinidad and Tobago (Auty and Gelb, 1986) and Bahrain (Katzman, 2010)—have long extracted and sold their fossil fuel reserves, as that brings in significant income. That process is also beginning in other SIDS, such as São Tomé e Príncipe (Frynas, Wood and Soares de Oliveira, 2003).

Timor-Leste is also aggressively pursuing its fossil fuel reserves, with the intention of extracting and selling oil and gas to generate income for development and nation-building (Steele, 2002). This strategy is being pursued although its contribution to the climate change problem will severely affect the country (that said, Timor-Leste is not legally obligated to reduce greenhouse gas emissions). This situation may reflect the limited understanding of climate change within the country and across ministries at the national level, or it might simply be a case of doublethink. A new but growing concern in Timor-Leste, climate change has elicited a slow response to developing effective laws, regulations, policies and plans to address the issue (Barbosa, 2006), yet it simultaneously enables the new state to pursue its development in a sustainable fashion. There is a clear lack of capacity not only for formulating relevant and applicable climate change-related laws, but also for ensuring these are enforced and that coordination takes place among relevant ministries and institutions (NCSA, 2007; NDES, 2007a; 2007b).

For example, forests play an essential role in climate change mitigation, as a carbon sink and in climate change adaptation, such as through ecosystem-based livelihoods and flood and landslide risk reduction.[10] Despite the banning of large-scale logging in 2000 and small-holder logging in 2008, logging continues on a minor scale, supported by widespread practices of slash-and-burn agriculture and the use of wood for fuel.

In addition, given the geology and steep terrain in Timor-Leste, loss of forest cover is a major contributory factor to worsening floods and landslides, the effects of which could be exacerbated by climate change with, for example, fewer but more intense rainfall events contributing to increased hazard impacts (Kirono, 2010). Some observers have identified deforestation as the key environmental issue in Timor-Leste (Sandlund et al., 2001), yet there are currently major discrepancies in estimates of forest distribution, cover and degradation and no forest inventory system exists (one is currently being developed). Without such systems alongside monitoring of natural resources in Timor-Leste, it is difficult to introduce laws and regulations on climate change, although climate change is one of many issues that need to be addressed.

## Disaster risk reduction and climate change adaptation in Timor-Leste: gaps and challenges

Integrating DRR, including CCA—plus climate change mitigation—into policy and planning for nation-building, as well as into relevant laws and legislation, is a long-term common goal of the NDMD, the UN Development Programme and other stakeholders, including civil society (NCSA, 2007; Norton and Waterman, 2008). This goal is an important contributory factor for Timor-Leste in building a resilient and sustainable state. However, the young country still faces gaps and challenges for DRR, including CCA. This section reviews current strengths, gaps and challenges in detail (see Table 4). Relevant and applicable lessons from other SIDS are provided with a view to supporting Timor-Leste in identifying appropriate ways forward. The contribution of each of these suggested actions to nation-building in Timor-Leste is then outlined.

### Vertical governance: coordination and communication

Timor-Leste's approach to DRR, including CCA, has tended to be top–down, with little local involvement or insufficient willingness and capacity to increase local-level involvement. A large disconnect exists between national and local-level governance, hindering coherent nation-building that is accepted at all levels. People become increasingly disenfranchised when their voices are not heard and underlying vulnerabilities are not addressed (Kelman, 2010; Wisner et al., 2004).

Adequate structures in Timor-Leste are already in place to effect improved vertical governance connections with the NDMD and Environment Secretariat at the top (national level) and the district, sub-district and *suco* disaster management committees at the bottom (local level). An active approach to DRR, including CCA, must adequately resource local-level disaster management committees through support from the national level (Hussain, 2008). The development of clear terms of reference as legally binding documents for all bodies involved would help clarify responsibilities and accountability for DRR, including CCA, in Timor-Leste and their subsequent contribution to nation-building.

The national level could support the development and the implementation of community-level plans. This would be achieved through the provision of guidance and direction in laws and regulations that support the development and implementation of DRR, including CCA activities, at the local level. National-level laws and regulations need to be flexible enough to allow for the context-specific nature of locally developed strategies (Lavell et al., 2012). This would then enable local-level planning to be incorporated and embedded into wider sub-district, district and national-level policies and plans. To integrate top–down and bottom–up approaches, national policies need to ensure that those who are most at risk are at the forefront of any strategy developed to reduce their risk (Weichselgartner and Obersteiner, 2002; White, Kates and Burton, 2001; Wisner, O'Keefe and Westgate, 1977). That involves local-level consultation with those most at risk at all stages, without one body dominating or controlling the other (Mercer, 2012).

While there has been continued rhetoric surrounding the need to link bottom–up and top–down strategies, it is rarely achieved in practice (Gaillard and Mercer, 2010). There are, however, excellent lessons to be learnt from other SIDS that could assist Timor-Leste in establishing an appropriate path for nation-building in a way that strengthens existing capacity (see Table 4).

Daly et al. (2010) highlight a specific mechanism used in addressing coastal management for Samoa, including climate-related hazards such as storm surge and climate change. The process of developing coastal management plans for the country's entire coastline was funded externally, was led by the national government and was based on local consultations in coastal villages following traditional practices for community meetings. District meetings helped to integrate the highly localised perspectives for addressing district-wide topics with which villages may not have connected on their own. The resulting local coastal management plans were integrated into a national coastal vulnerability reduction strategy. The participation process was facilitated by external consultants but was used as an opportunity to train national and local staff in participatory methods, so that the coastal management plans could be regularly reviewed and updated, rather than being approved and then remaining static.

Samoa's approach could be emulated to produce coastal management plans and vulnerability reduction strategies for Timor-Leste, thereby better connecting vertical governance and thus contributing to nation-building tasks. Guidance and facilitation from the national level would thus lead to local decision-making, which is subsequently integrated at wider scales. The goal is to strengthen local power and control for DRR, including CCA, without dismissing the guidance needed from the national government. By creating such local–national connections and trust, Timor-Leste's government can gain credibility across the country without being overly controlling, thereby significantly contributing to nation-building.

A further example comes from Tikopia and Anuta, small islands in the far eastern Solomon Islands. The islands have neither airstrips, nor jetties, nor reliable off-island communication systems. On 28 December 2002, Tikopia and Anuta were struck by Category 5 Cyclone Zoë (Yates and Anderson-Berry, 2004). No one on the two islands died immediately because the people knew the impending signs of a coming storm and how to respond. Some of their traditional housing survived—along with fermented breadfruit that they buried in the ground in case of cyclones, although finding the buried fruit proved to be troublesome (Treadaway, 2007). The population had retreated to higher ground and stayed under overhanging rocks, thereby avoiding the cyclone-related flooding while being sheltered from the high winds. That exemplifies bottom–up actions for disasters by SIDS people. The inhabitants of the small islands had their own knowledge regarding a coming cyclone and how to survive it.

The flipside is that little food and water survived the storm, leaving the islanders in need of emergency assistance. Their radios for off-island communication had not worked before the storm, so there were no means to communicate about their situation. The outside world did little to assist until a journalist hired a helicopter in nearby Vanuatu, landed on one of the islands and brought the story to the world by selling an exclusive to an Australian newspaper. Eventually, a ship with needed relief supplies arrived. That exemplifies top–down actions for disasters. A disaster-affected population needed external help, which—after an unnecessary delay—was provided by authorities far away.

By learning the lessons from Samoa and the Solomon Islands—as well as similar initiatives from SIDS that combine top–down and bottom–up approaches (Cronin et al., 2004; Kelman, Mercer and West, 2009; Mercer et al., 2009; 2010)—Timor-Leste could support communities in dealing locally with DRR, including CCA, without sacrificing national unity. No expectation should be created that the national government will attend to all DRR, including CCA, and disaster response needs. In fact, even affluent countries such as the United States and Australia advise communities to expect to be on their own for at least 72 hours after a major disaster (EMA, 2003; FEMA, 2004). That is in line with disaster studies, which frequently articulate the importance of locally driven and managed approaches for DRR, including CCA (Wisner et al., 2004; Reid et al., 2009).

## Horizontal governance: coordination and communication

Coordination and communication across line ministries at the national level needs to be strengthened with clearly defined channels of communication. Poor coordination and communication at the national level directly impacts the ability of those at the local level—that is, district authorities—to work with communities to implement effective DRR, including CCA measures. As outlined above, the absence of specific legal frameworks and laws defining the responsibilities of individual line ministries and district administration offices in terms of DRR, including CCA, has ultimately rendered the NDRMP ineffective.

A similar scenario evolved throughout the development of Timor-Leste's NAPA. While the production of the document involved joint meetings and discussions with key government officials from different line ministries—including the NDMD and the Environment Secretariat—it was again facilitated and coordinated by external experts. This occurred as a direct and urgent response to the need for Timor-Leste to meet international obligations under frameworks such as the UNFCCC and the UN International Strategy for Disaster Reduction (UNISDR). As a consequence, the process of meeting these obligations does not contribute to long-term sustainability and nation-building for Timor-Leste. Government employees are not empowered to take ownership of resulting documents or frameworks, nor do they learn the governance skills necessary to implement such documents across line ministries (NCSA, 2007).

Further capacity and human resources are also needed at the district level in order to provide support to communities in the form of technical knowledge, which could link to local knowledge of hazards and vulnerabilities. This would empower communities to consolidate their existing approaches and to integrate these with relevant and applicable outside knowledge that further contributes to DRR, including CCA.

To help facilitate coordination and communication at a horizontal level, DRR, including CCA, 'champions'—those with the skills, training and interest in the topic—could be situated within each line ministry (and at the district level) with the responsibility to ensure that DRR, including CCA, is understood and taken into account by the respective line ministry and associated laws, regulations and policies. That would create horizontal governance connections, with the aim of reducing duplication and of encouraging that efforts of one ministry do not run counter to those of other ministries, thereby contributing to good governance and development tasks for nation-building.

Such a process needs to be supported by a strong directive from the top, stressing the importance of DRR, including CCA, to Timor-Leste and its nation-building (Ahrens and Rudolph, 2006)—in essence linking horizontal and vertical governance. Without clearly defined governance structures that are accountable, transparent, clear and coherent— especially in terms of laws and regulations—and that include participation from and communication among sectors, disaster risk is likely to continue manifesting as disasters and to impinge on nation-building (Ahrens and Rudolph, 2006; GNDR, 2011; O'Brien et al., 2012). For example, as Hussain (2008) outlines, while an effective governance system is essential for vulnerability reduction in SIDS, the success of such a system depends on the education and capacity of those employed within it. Linking Timorese government officials with those in other SIDS and supra-national institutions could help build and support the capacity of the Timorese government in terms of these key issues through coordination and communication, both horizontally and vertically.

## Large donor presence

In terms of connecting DRR, including CCA, to nation-building, Timor-Leste is both helped and hindered by the large international donor presence within the country. Issues of ownership, aid dependency, inertia, hand-out mentalities and diverse and sometimes conflicting interests of donors and recipient countries all need to be carefully considered (Bertram and Watters, 1985; Feeny and McGillivray, 2010; Tuiloma-Palesoo, 2004). In 2010, Timor-Leste had an annual aid budget of $256.8 million (Ministry of Finance, 2010). Such a large aid budget also presents potential for donors to inadvertently fuel corruption and create excessive administrative burdens within Timor-Leste, activities that are detrimental to nation-building. Such problems could potentially increase, given expected future opportunities to access large international funds for climate change adaptation and mitigation. Timor-Leste has started to take steps to address these challenges through the Timor-Leste Transparency Portal (n.d.) and by ensuring that donor agencies are aligned with government priorities.

Timor-Leste's situation is not unusual. SIDS are recipients of some of the highest relative levels of development assistance, compared to their economies and populations (Feeny and McGillivray, 2010). In a study of the impacts of foreign aid on SIDS, Feeny and McGillivray (2010) suggest that while aid is generally effective at increasing economic returns, overly high levels of aid may not be effective beyond the recipient SIDS' ability to absorb the aid.

Aid can thus have positive and negative effects. Donors have the potential to create dependency given the tendency of recipient countries to sit back in the knowledge that donors will come to their rescue, as has occurred within Pacific SIDS. It should be the responsibility of donors to work in partnership with recipient country governments to minimise negative impacts. For example, donor agencies could provide financial and moral incentives, rewarding countries that take early action as opposed to waiting for support (Bettencourt et al., 2006). In addition, in the presence of large donor numbers, there are added complications in terms of coordination, communication and duplication of effort (NCSA, 2007). Moreover, donors compete to align themselves politically and to support Timor-Leste as a newly established independent state.

## Data availability: hazard and vulnerability

A specific challenge for nation-building in Timor-Leste is the lack of credible data sets of past disasters and environmental history, including weather and climate data (Barbosa, 2006; Barnett, Dessai and Jones, 2007). This is a result of data not being collected, destroyed due to conflict or spread across archives in Australia, Indonesia, Portugal and Timor-Leste. As outlined above, the NDMG and Pacific Climate Change Science Program are attempting to address this issue. Yet a wealth of knowledge available within the country has not yet been tapped into: local knowledge.

Increasingly, there is a wider international focus on the benefits of local knowledge for DRR, including CCA (Dekens, 2007a; 2007b; Kelman, Mercer and Gaillard, 2012; Shaw, Sharma and Takeuchi, 2009; Shaw, Uy and Baumwoll, 2008). Even if there were no lack of documented scientific data in Timor-Leste, local knowledge should be taken into account (Agrawal, 1995; Baumwoll, 2008; Gaillard et al., 2008; Mercer, 2012). Local knowledge is constantly evolving and developing through contact with external systems and knowledge (Flavier, de Jesus and Navarro, 1995). This fluidity and ability to integrate with other knowledge forms needs to be capitalised on to identify the most appropriate and effective strategies, irrespective of knowledge origins, for DRR, including CCA, in relation to nation-building. If undertaken with local communities, a countrywide consultative, participatory assessment of disaster risk, including climate-related risk, could help to bridge data gaps and thereby support relevant policy and action for DRR, including CCA, across sectors in Timor-Leste. It would also enhance local power, ensuring that Timorese people feel that they were part of creating the Timorese state and contributing towards the development of its national identity.

Such an approach was taken in the Maldives. The Indian Ocean tsunamis of 2004 impressed upon the Maldives the need for comprehensive risk analysis; the completed assessment identified the most vulnerable and risk-prone areas (UNDP, 2006). This information has since been used to help inform the identification and implementation of coping and adaptive strategies for communities at risk as well as development planning and support of the government in reducing disaster risks—all within the context of this SIDS' new democracy. The Maldives now has a fully integrated planning and coordination system linking DRR, CCA and development (Lavell et al., 2012).

Timor-Leste could learn from such work in contributing to its own nation-building, which starts with understanding hazards and vulnerabilities. That links to improving horizontal and vertical governance, as noted above.

## Integrating disaster risk reduction and climate change adaptation

At the national level in Timor-Leste, DRR and CCA currently operate in relative isolation from each other and from other related sectors, such as agriculture, health, infrastructure and education. At the local level, broader integration of DRR, including CCA, with other sectors is starting to occur (Oxfam, 2011). For example, international non-governmental organisations (NGOs) are working in partnership with local NGOs, local government and communities to identify levels of risk to hazards, including climate change, and to develop action plans to reduce identified risks. This approach is empowering local communities and authorities to address their risks themselves.

To contribute to unity and to wider nation-building, a nationwide, coordinated approach must enable all risks to be addressed within one framework that is owned and supported by all relevant stakeholders. If CCA were embedded within DRR,[11] it would enable the current national and local government structures for DRR within Timor-Leste to be applied for factoring in climate change[12]—again supporting resolution of the horizontal and vertical governance challenges discussed above. DRR, including CCA, also needs to be placed within wider development and sustainability contexts to maintain the processes over the long term (Lewis, 1999; Wisner et al., 2004).

Dealing with disasters, including the problems arising from climate change, is a key challenge for SIDS, but it is not the only development and sustainability challenge (Lewis, 1999; Méheux, Dominey-Howes and Lloyd, 2007; Mercer et al., 2007;

UN, 1994; 2005). Research has made a forceful case that DRR must be viewed within wider contexts rather than separated out as a distinct sector (Lewis, 1999; Shaw, Pulhin and Pereira, 2010a; 2010b; and Wisner et al., 2004). That includes incorporating CCA, whose activities should be enacted for DRR anyway (Shaw, Pulhin and Pereira, 2010a; 2010b).

An additional problem for Timor-Leste as a young state reeling from many years of conflict is overdependence on external experts and consultants due to a lack of internal capabilities (NDES, 2007a; 2007b). A resulting outcome from these short-term missions is often a lack of ownership of the documents produced or a feeling of disempowerment of those involved. Consequently, outputs do not become widely adopted at either the national or the local levels. Due to its geographical location, Timor-Leste to date is not directly involved in, and has limited access to, supra-national institutions that support SIDS in other regions, such as the Secretariat for the Pacific Community's Applied Geoscience and Technology Division, the Secretariat for the Pacific Regional Environment Program, the Caribbean Community Climate Change Centre and the Caribbean Disaster Emergency Management Agency.

Given its close proximity to the South Pacific, Timor-Leste could join the Pacific SIDS' institutions. Many Strong Voices, or MSV, is one programme that can provide related assistance (MSV, n.d.; CICERO and UNEP/GRID-Arendal, 2008; Kelman and West, 2009). MSV brings together people, organisations and institutions from SIDS as well as the Arctic to address climate change in the context of sustainability and development. CCA and climate change mitigation are explicitly included, but within the context of community sustainability. Many participants come from autonomous SIDS and non-SIDS jurisdictions, such as the Cayman Islands, the Cook Islands, Greenland and Nunavut. Even without full independence, the governments of places such as Greenland and Nunavut have increased levels of powers, so that they are, to some extent, undergoing a nation-building process. Within this political context, MSV aims to support the voices of these small, remote places in international forums, without losing the need for or focus on local interests, voices and governance. The participants recognise the immense challenge that climate change brings, but also the need to view climate change in context and to link it to DRR and other development processes (Kelman, 2010).

One practical area in which that is happening is ecosystem-based DRR, including CCA, whereby the management and preservation of ecosystems for livelihoods also implements DRR, including CCA (PEDRR, 2010; Sudmeier-Rieux and Ash, 2009). A method called Risk and Vulnerability Assessment Methodology Development Project was piloted in the SIDS of Jamaica (UNEP, n.d.; 2010). Not unlike the Samoan experience described by Daly et al. (2010), the project was externally supported but engaged national-level partners to consult with local communities, using traditional approaches to understand how ecosystems and livelihoods could be better connected to maintain both through DRR, including CCA.

Timor-Leste could implement a similar method, tailored to the country rather than to Jamaica, in order to connect sustainable livelihoods, environmental management and DRR, including CCA. These processes would contribute to nation-building by letting Timorese set their own agenda and help themselves. The idea, in theory, is that national leadership would lend coherence to the country while supporting local empowerment, as per MSV. That is, the national government would use the process of DRR, including CCA, to support skills and actions at the local level, while ensuring that local approaches do not create or exacerbate problems elsewhere. That becomes nation-building as the local level takes charge of livelihoods and environmental management, inspired by the national level and with national-level monitoring to ensure countrywide coherence. Timor-Leste has the structures and resources in place to apply this method locally. It remains to be seen whether it will be completed.

### Summary

This section has outlined gaps and challenges in the context of DRR, including CCA, for nation-building in Timor-Leste and has provided examples from other SIDS from which Timor-Leste could learn in developing appropriate policies and actions. These are summarised in Table 4.

## Conclusion: possible trajectories

Few examples exist of direct implementation of DRR, including CCA, as part of nation-building. Indeed, Timor-Leste's situation is relatively unique, but the country can learn, in particular, from experience in other SIDS that are working on DRR and CCA. That should cover not only vulnerabilities that cause problems in the country, but also the resiliencies that provide ways of improving. In trying to use DRR, including CCA, to contribute to nation-building, challenges and opportunities emerge. Moreover, DRR, including CCA, is only one part of a wide array of development and sustainability challenges facing Timor-Leste. Other examples are unsustainable farming and fishing practices, soil erosion and an overdependence on unsustainable oil and gas exploitation (Scheiner, 2011). This is in addition to social issues including coming to terms with past violence, reducing gender inequity and dealing with gender-based violence (Nevins, 2003; Hynes et al., 2004).

In addition, political stability is not a given. In February 2008, Timor-Leste's president and prime minister were nearly assassinated in a coup attempt. The result was that the rebel leader, Alfredo Reinado, was killed and his group effectively finished, but that does not prevent further dissatisfied factions from seeking violent means (Arnold, 2009). Timor-Leste must also deal with the challenge of governing its exclave and one inhabited outlying island, with many examples throughout history demonstrating the political instability that physically separated jurisdictions can bring (Baldacchino and Milne, 2006; 2009; Vinokurov, 2007). In small countries, political and personal factions within ministries and agencies are not uncommon and can be serious obstacles to moving forward, especially in a young country struggling to establish itself as an independent state.

**Table 4** DRR, CCA and nation-building in Timor-Leste: strengths, challenges, opportunities and lessons

| | Current strengths for DRR, including CCA | Current gaps and challenges for DRR, including CCA | Suggested trajectories | Applicable lessons from other SIDS | Contribution to nation-building |
|---|---|---|---|---|---|
| **Vertical governance: coordination and communication** | • Decentralised disaster risk management structure in place.<br>• NDRMP and NAPA developed.<br>• NDMD and DDMCs working with civil society to implement community-based disaster risk management.<br>• A number of donor-led DRR and CCA programmes.<br>• Civil society providing training for decentralised DRR structures. | • NDRMP not fully understood.<br>• No national-level laws and regulations.<br>• Top–down framework of governance with little local involvement.<br>• Human and financial resources inadequate.<br>• Communication between local-level disaster management commissions and NDMD not regular or reliable.<br>• Lack of accountability and transparency. | • Key ministerial officials identified to provide permanent links between national and local levels.<br>• Time and resources allocated for local-level consultation.<br>• National-level laws and regulations developed from local-level consultations.<br>• Laws flexible enough to include localised issues and responses.<br>• Proactive rather than reactive approach linking bottom–up and top–down. | • Response to Cyclone Zoë in the Solomon Islands (Yates and Anderson-Berry, 2004).<br>• Integrated coastal management in Samoa (Daly et al., 2010).<br>• Multi-stakeholder participation in the Solomon Islands (Cronin et al., 2004).<br>• Linking local and scientific knowledge in Papua New Guinea (Kelman, Mercer and West, 2009; Mercer et al., 2009; 2010). | • Increased trust and local–national connections lead to increased credibility of Timor-Leste's government.<br>• Working in partnership and empowering communities helps to gain their support and increase national unity.<br>• National support for the building of local capacity in DRR, including CCA, will lessen the impact of future hazards. |
| **Horizontal governance: coordination and communication** | • NDMD established.<br>• Links with other line ministries visible.<br>• NDRMP and NAPA developed. | • CIGD not working as lack of interest from individual ministries and no strong top–down direction.<br>• No ministerial DRR and CCA champions driving the work forward.<br>• No ownership by relevant bodies of NAPA and NDRMP documents, which are poorly understood.<br>• Inadequate resources. | • Decision to embed CCA and mitigation within a DRR framework.<br>• Priority given to DRR, including CCA, and strong direction from top-level government required.<br>• Identification of key DRR and CCA 'champions' and of key bilateral inter-ministerial relationships for lobbying.<br>• Refocusing and provision of top–down support for CIGD—lobbying of key actors, such as the prime minister, vice prime minister and key ministers to raise political importance of DRR, including CCA.<br>• Review of the process and systems at the national level for DRR including CCA.<br>• Legally binding instruments clarifying responsibilities and accountability for CIGD members. | • Learning from networks established among Pacific and Caribbean SIDS.<br>• Cuba's governance tends to work well across sectors at each governance scale for hurricane evacuation (Thompson and Gaviria, 2004; UNDP, 2007), although it should be kept in mind that the country is a dictatorship. | • An accountable, transparent, coherent and participative governance structure would enhance nation-building.<br>• Increases education and capacity building of citizens.<br>• Increases the sustainability of initiatives, thus contributing to nation-building in the long term.<br>• Establishes a positive example of how inter-ministerial coordination and communication can be undertaken—a model for other sectors. |

| | Current strengths for DRR, including CCA | Current gaps and challenges for DRR, including CCA | Suggested trajectories | Applicable lessons from other SIDS | Contribution to nation-building |
|---|---|---|---|---|---|
| **Large donor presence** | • Large donor interest and support provided to Timor-Leste. | • Uncoordinated and poor communication within and between donors and national government.<br>• Duplication of activities.<br>• Competition for resources.<br>• Extensive use of short-term consultants.<br>• Inadequate funding from the national budget for DRR, including CCA. | • Embedding of international obligations within national frameworks.<br>• Capacity building of Timorese; knowledge building in different sectoral ministries.<br>• Linking with and learning from wider networks.<br>• Agencies and staff to develop key skills and specialisations.<br>• Donor agencies ensuring application processes are collaborative rather than competitive, avoiding breeding an environment of secrecy.<br>• Grants established and approved in line with Timor-Leste's government plans, promoting effectiveness.<br>• Meeting effectiveness: good meetings often lead to good collaboration and motivation. | • No examples of successful donor coordination in a SIDS were found in the scientific literature, with practitioners still citing donor coordination as being a major challenge for SIDS (MacLellan, 2011). | • The approach to DRR, including CCA, is structured and coordinated.<br>• The duplication and replication of efforts is avoided.<br>• The benefits of DRR, including CCA strategies, are realised in the long term.<br>• Aid effectiveness is ensured for DRR, including CCA. |
| **Data availability: hazard and vulnerability** | • Data is available but it is not consolidated.<br>• A large amount of untapped data is available in the form of local knowledge. | • Lack of or loss of credible data sets.<br>• Existing data spread across archives in Australia, Indonesia, Portugal and Timor-Leste in different languages.<br>• No existing data bank or library of data. | • Use of relevant and applicable local and scientific knowledge.<br>• A countrywide consultative, participatory assessment of disaster risk.<br>• Development of relevant policy and action. | • Comprehensive risk analysis in the Maldives (UNDP, 2006).<br>• Research on climate knowledge and vulnerability assessment in Timor-Leste (Oxfam, 2011).<br>• Pacific Climate Change Science Program (Da Silva and Moniz, 2010). | • Comprehensive information and a data bank are available to inform appropriate nation-building activities.<br>• Local power is enhanced in order to contribute to the creation of a national identity. |
| **Integrating disaster risk reduction and climate change adaptation** | • Partnerships between NGOs, local government and communities established at local levels.<br>• Structures and resources already in place. | • Sectors operating in isolation.<br>• Issues not taken seriously within and across line ministries.<br>• Overdependence on external experts.<br>• Lack of curriculum development for DRR, including CCA, in higher education. | • A countrywide coordinated approach.<br>• Join SIDS networks especially in the South Pacific to learn and share knowledge.<br>• Embedding CCA within DRR frameworks.<br>• Ecosystem-based DRR, including CCA. | • Risk and vulnerability assessment methodology in Jamaica (UNEP, 2010).<br>• Many Strong Voices programme (CICERO and UNEP/GRID-Arendal, 2008). | • National leadership lends coherence to the country while empowering local action.<br>• Local communities and authorities are empowered.<br>• Timorese able to set and direct their own agenda. |

**Source:** authors.

Addressing such issues takes time, yet Timor-Leste has the opportunity to draw on the world's detailed contemporary history of nation-building. That includes the break-up of the Soviet Union in 1991. The three Baltic republics quickly established stable democracies, revitalised their economies and are now members of the European Union. In contrast, the Central Asian republics tended towards repressive dictatorships rife with corruption and unregulated pollution. Timor-Leste has the advantage of a comparatively stable democracy, donors committed to sustainable environmental management (for the moment) and a process that embraces traditional and indigenous approaches for nation-building (Nixon, 2008).

Timor-Leste can also draw on its SIDS networks, learning from DRR and CCA successes and failures around the world. Emulating Samoa's process for developing coastal management plans (Daly et al., 2010) would be an excellent start. It would also be useful to put in place measures—such as the transparency portal—to prevent the type of corruption and nepotism that plagued Antigua and Barbuda until 2004, when a family dynasty was defeated in a national election (Coram, 1993; Erikson and Minson, 2005). Learning from Nauru's failure to manage its natural resource wealth (Connell, 2006; Gowdy and McDaniel, 1999) would also help to avoid similar problems.

Ultimately, Timor-Leste has plenty of support and opportunities for the young country to select its own trajectory out of underdevelopment. That means tackling the various sustainability and nation-building challenges simultaneously, in a way that supports both rather than trading them off against each other. The lure of quick money for development through the exploitation of natural resources, including fossil fuels, or through dependence on a single donor, such as China, are major hurdles to overcome (La'o Hamutuk, 2011). In Timor-Leste's nation-building work, DRR, including CCA, should neither dominate nor be neglected. Rather, it should be incorporated and delivered through key nation-building tasks, including governance and development, to ensure a sustainable path for nation-building in Timor-Leste while avoiding potential detrimental effects from hazards such as climate change. If that were achieved, then Timor-Leste would become a lesson to the world, especially small countries, on how to incorporate DRR, including CCA, into nation-building.

## Acknowledgements

The authors would like to thank all stakeholders involved in this research, including members of local communities in Timor-Leste who expressed their opinions regarding DRR and CCA governance, local and national government colleagues who provided significant insight and feedback, local civil society and representatives of international NGOs who have been involved with DRR, including CCA, in Timor-Leste. They extend special thanks to AusAID. The views expressed in this paper do not necessarily reflect those of the stakeholders, including the Australian Department of Foreign Affairs and Trade, the Government of Australia and the Government of Timor-Leste.

## Correspondence

Jessica Mercer, Secure Futures Consultancy, Winchester, Hampshire, United Kingdom. E-mail: jessica@secure-futures.net; jessica-mercer@hotmail.com.

## Endnotes

1 Jessica Mercer is Director at the Secure Futures Consultancy, Winchester, Hampshire, United Kingdom; Ilan Kelman is Reader at the Institute for Risk and Disaster Reduction and Institute for Global Health, University College London, London, United Kingdom, and a Senior Research Fellow at the Norwegian Institute of International Affairs, Oslo, Norway; Francisco do Rosario is Director of the National Disaster Management Directorate, Dili, Timor-Leste; Abilio de Deus de Jesus Lima is Secretary of State for the Environment in the Ministry of Economy and Development, Dili, Timor-Leste; Augusto da Silva is National Technical Advisor to the Secretary of State for the Environment, Ministry of Economy and Development, Dili, Timor-Leste; Anna-Maija Beloff is Regional Disaster Management Coordinator for the Caribbean, International Federation of Red Cross and Red Crescent Societies, Trinidad and Tobago; and Alex McClean is Manager at Arid Edge Environmental Services, Alice Springs, Australia.

2 For further information on how each of these components should be organised and employed, at what levels, time periods and cost, see Dobbins et al. (2007).

3 See, for example, Chernilo (2007) and Dobbins et al. (2007).

4 See, for example, Ferris (2011) and UNDP (2008).

5 See, for example, Lewis (1990); Méheux, Dominey-Howes and Lloyd (2007); Mercer et al. (2007); Mossler (1996); Pelling and Uitto (2001); and Turvey (2007).

6 See, for example, Daly et al. (2010); Gero, Méheux and Dominey-Howes (2011); UN (1994; 2005); UNISDR (2005).

7 For further information on the 2006 crisis, see Kingsbury and Leach (2007) and Scambury (2009).

8 See, for example, Bosher and Coaffee (2008) and Manyena et al. (2011).

9 See, for example, Lape and Chin-Yung (2008).

10 For more information on forests and climate change in Timor-Leste, see Godinho et al. (2003).

11 See also Shaw, Pulhin and Pereira (2010a; 2010b).

12 See also Gero, Méheux and Dominey-Howes (2011) for a discussion of Pacific SIDS.
